# Photoperiod may regulate growth via leptin receptor A1 in the hypothalamus and saccus vasculosus of Atlantic salmon (*Salmo salar*)

**DOI:** 10.1080/19768354.2019.1595138

**Published:** 2019-04-12

**Authors:** Liang Chi, Xian Li, Qinghua Liu, Ying Liu

**Affiliations:** a Qingdao Agricultural University, Qingdao, People’s Republic of China; b Institute of Oceanology Chinese Academy of Sciences, Qingdao, People’s Republic of China

**Keywords:** Photoperiod, leptin receptor, growth, Atlantic salmon

## Abstract

Photoperiod is believed to regulate growth in fish, although the mechanism involved is still unclear. In this paper, we report a relationship between leptin-receptor A1 (*AsLRa1*), melatonin-receptor (*AsMR*) and photoperiod in Atlantic salmon. Atlantic salmon (mean weight 1071.70 ± 155.54 g) were reared under six photoperiod regimes, four constant light regimes 24L:0D, 18L:6D, 12L:12D and 8L:16D, hours of light (L) and dark (D) and two varying light regimes, LL-SL = 24L:0D-8L:16D, and SL-LL = 8L:16D-24L:0D over a period of seven months. The results showed that *AsLRa1* transcripts were mainly existed in the hypothalamus and saccus vasculosus (SV), *AsMR* was mainly expressed in the hypothalamus. Long photoperiod inhibited the expression of *AsLRa1* and *AsMR* transcripts in the Atlantic salmon brain. The expression pattern of *AsLRa1* was similar to the expression pattern of *AsMR* in the hypothalamus. Food intake was higher in fish with lower *AsLRa1* transcript levels. This demonstrated that photoperiod influenced somatic growth by changing expression of *AsLRa1* in the hypothalamus and SV to affect appetite. In addition, we found that the SV appears to act as a seasonal sensor regulating reproduction in a similar way to the hypothalamus.

## Introduction

Environmental factors (e.g. day length, temperature, oxygen availability, rainfall, etc.) play important roles in regulating physiological function, including reproduction and growth in fish (Boeuf and Le Bail 1999; Shin et al. 2014). Among these environmental factors, only day length (photoperiod) shows periodicity with seasonal changes, which is crucial to determine the timing of reproduction and growth. Many researchers have found that photoperiod could affect growth of fish. Such as Biswas et al reported that extended and continuous photoperiods could significantly improve the growth performance of striped knifejaw (*Oplegnathus fasciatus*) (Biswas et al. 2016). Atlantic salmon (*Salmo salar.* L) also displays seasonal changes in growth (Forsberg 1995; Kadri et al. 1997), and as a consequence, their growth could be affected by day length or artificial light (Endal et al. 2000; Smith et al. 1993). Atlantic salmon exhibit increased growth rate under continuous light during winter compared with fish under to a natural photoperiod(Duncan et al. 1999; Kråkenes et al. 1991; Oppedal et al. 2001; Porter et al. 1999). Furthermore, light–dark (LD) transitions are also important in synchronizing locomotor activity rhythms. Feeding activity mainly appears during the day, meanwhile, diet rhythms are affected strongly by LD cycles in Atlantic salmon and rainbow trout (*Oncorhynchus mykiss*) (Iigo and Tabata 1997; Jones et al. 2002), suggesting that day length could modify growth by increasing food intake indirectly (Boeuf and Le Bail 1999). Atlantic salmon are sensitive to photoperiod, and some studies demonstrated that food intake and food conversion efficiency are directly correlated and generally highest with increasing photoperiod (Berg et al. 1992). In conclusion, a long constant or increasing photoperiod promotes salmon growth. However, the mechanism of photoperiod influencing growth in fish is still not fully understood.

Leptin is secreted by adipose tissue and has an important role in regulating appetite, adiposity, food intake and energy expenditure in mammals (Fuentes et al. 2013; Macdougald et al. 1995; Schwartz et al. 2000). Leptin interacts with several neuropeptides to regulate food intake in the hypothalamus (Minokoshi and Kahn 2003). The physiological functions of leptin are mediated by the leptin receptor (LR) in mammals (Bates et al. 2005). In fish, leptin or LRs have been identified in many species including zebrafish, medaka, Arctic charr, rainbow trout and Atlantic salmon (Froiland et al. 2010; Gorissen et al. 2009; Kurokawa et al. 2005; Murashita et al. 2008; Ronnestad et al. 2010). The functions of leptin and LRs in fish are similar to those in mammals. The action of leptin is mediated through LRs expressed on appetite-related neurons and circuits in the hypothalamus (Liu et al. 2010). Studies in mammals have found that the expression of leptin is rhythmic, which is related to the pineal melatonin axis in ruminants (Klocek-Gorka et al. 2010; Zieba et al. 2007; Zieba et al. 2008). In fish, the rhythm of leptin/LRs is mainly focused on the feeding regime. Daily changes in leptin mRNA were first studied in Atlantic salmon, in which changes were seen in white muscle, belly flap, visceral adipose tissue and liver, when fish are exposed to short term feeding restrictions (Moen and Finn 2013). Meanwhile, in goldfish, hepatic leptin expression peaks appear at 9 h post feeding (Tinoco et al. 2012). These results suggest that leptin/LRs could be affected by environmental factors. Up to now, however, our understanding of the relationship of leptin/LRs and photoperiod is still limited.

Photoperiod could regulate growth in fish and growth is related to leptin and LRs, thus, we hypothesized that there may be some relationship between photoperiod and leptin/LRs. Atlantic salmon, are native to the North Atlantic and its surrounding rivers, and were introduced into China using Recirculating Aquaculture Systems (RAS). In our previous study, we found that photoperiod significantly affected growth of Atlantic salmon reared in a RAS. In this paper, the relationship between photoperiod and leptin/LRs of Atlantic salmon were investigated using a RAS. In addition, the regulation center for photoperiod is mainly located in the brain, therefore, this study focused on Atlantic salmon LRs, which are also mainly expressed in the brain.

## Materials and methods

### Experimental design

Atlantic salmon (weight: 1071.70 ± 155.54 g) were collected from Shandong Oriental Ocean Sci-Tech Co. Ltd., Shandong province, China. The fish were randomly distributed into experimental RAS tanks (130 cm high × 200 cm diameter) and reared by satiation feeding with a commercial salmon diet (Skretting, Norway) containing 48% protein and 18% fat twice daily during the period of light manipulation, and the total food consumption of each tank was recorded. Each experimental tank contained ∼60 fish. The water temperature was maintained at 16.27 ± 0.54°C, total ammonia-nitrogen < 0.25 mg/L, salinity 24–26 and a pH between 7.2 and 7.5.

Six photoperiod treatment groups were designed. Four of the photoperiods were constant throughout the experiment 24L:0D, 18L:6D, 12L:12D and 16L:8D [hours of light (L) and dark (D)]. The other two photoperiods varied. In the first, the photoperiod changed from 24L:0D to 8L:16D (LL-SL treatment), and in the second changed from 8L:16D to 24L:0D (SL-LL treatment), with the lighting period changing 5 min per day in both cases. Each group contained three replicate tanks (60 fish/tank). The experiment was performed from September to the following March, a period spanning the first reproductive period of Atlantic salmon. First, all fish in each tank were anesthetized using 0.05% MS-222 and measured for body weight and body length every month individually. Then fish were sampled, and three fish in each tank were anesthetized to death in seawater using 0.05% MS-222. Body mass and length were recorded. The gonads were stored in Bouin’s fixative for 24 h and then in 70% ethanol for histological examination to confirm the specific stage of the experimental fish. All of the procedures described in this study were reviewed and approved by the ethical committee of the Institute of Oceanology, Chinese Academy of Sciences.

The feeding ratio (FR) was calculated as FR (%) = 100 × F/[0.5 × (BW_2_ + BW_1_) × (T_2_-T_1_)], where BW_1_ and BW_2_ were the average individual weight at days T_1_ and T_2_. F was total food consumption.

### RNA preparation, synthesis of first-strand cDNA and quantitative real-time PCR

The brain were isolate from encephalocoele using RNAase-free bone shears and tweezers, after that, the whole brain were washed 3 times in RNAase-free PBS. Then the each part of brain [Telencephalon, Diencephalon, Hypothalamus (hypothalamus is located on the under surface of diencephalon and on the top of pituitary), Mesencephalon and saccus vasculosus (SV) (SV is a red saccus located on the back of medulla, and it’s the only red organ in fish brain)] were separated carefully using tweezers and scalpel and stored in Liquid nitrogen immediately. Total RNA were extracted from the different regions of the Atlantic salmon brain using a fast 200 RNA extraction kit (Fastagen, Shanghai, China), according to the manufacturer’s instructions. Total RNA were dissolved in 20 μL RNase-free water. Then, 2 μg RNA was reverse transcribed to first-strand cDNA by a First- Strand cDNA Synthesis SuperMix (TransGen, Beijing, China). The reaction system contained 1μL genomic DNA remover, 0.5 μL Oligo dT Primer, 10 μL of 2 × TS reaction mix and RNase-free water up to a volume of 20 μL.

Quantification of *AsLRa1* and *AsMR* gene expression was carried out with SYBR *TransStart* Top Green qPCR SuperMix Kit (*TransGen*, Beijing, China) using the standard curve method with *β-actin* as a reference gene and performed in an Eppendorf Mastercycler ep realplex real-time PCR instrument (Eppendorf, Germany). The primers used to amplify *AsMR*, *AsLRa1* and *β-actin* are listed in Table 1. Amplification was performed in a 20 μL reaction volume according to the manufacturer’s instructions, using 0.4 μL Passive Reference Dye, 10 μL 2×Top Green qPCR SuperMix, 1 μL cDNA, 0.4 μL (4 μM) forward and reverse primers and deionized distilled water up to a final volume of 20 μL. The qPCR programs were performed as follow: 94°C for 30 s followed by 40 cycles of 94°C for 5 s, 60°C for 15 s and 72°C for 10 s followed by a temperature ramp for melting curve analysis.

**Table 1.** The primers used for Real-time RT-PCR.

Genes	Sequences of primers	Products (bp)
*AsMR*	F: 5’-GCAACTTGCTGGTTATCATTTCAGTG-3’	245
	R: 5- GTGACAGATGTAGCAGTAGCGGTTG-3	
*AsLR*	F:5’-GCTTATGATCCGCCTTTGAATTTGTG-3’	284
	R:5’-CTCGGTCTTCTTCCTTTCCTCTGTTG-3’	
*β-actin*	F: 5’-ATCCACGAGACCACCTACAACTCC-3’	268
	R: 5’-CGTACTCCTGCTTGCTGATCCAC-3’	

Note: F: forward primer; R: reverse primer.

### *In situ hybridization* of Atlantic salmon brains

The brains of Atlantic salmon were fixed in 4% paraformaldehyde in 0.1 M PBS (phosphate buffered saline, pH 7.4) overnight at 4°C. The samples were dehydrated in a graded series of methanol. Sections of paraffin-embedded brains were prepared on 5μM glass slides coated with 0.1% poly-L-lysine solution. The partial CDS of *AsMR* and *AsLRa1a1* were cloned into pGEM-T vectors for preparing sense and antisense RNA probes from a T7 or SP6 promoter by using FITC or digoxigenin (DIG) RNA Labeling Kit (Roche) respectively (Table 2). The sections were hybridized with the sense or antisense probes at 66°C for 18 h. After hybridization, the samples were incubated overnight at 4°C with horseradish peroxidase (HRP)-conjugated anti-FITC–antibody (Roche) at a 1:2000 dilution in blocking solution to detect the FITC signal. After three washes in PBST, the samples were incubated 1 h in tyraminde signal amplification (TSA)-fluorescein at a 1:150 dilution in TSA buffer. The DIG signal was detected in samples. They were incubated overnight at 4°C with HRP-conjugated anti-DIG antibody (Roche) at a 1:2000 dilution in blocking buffer with 1% H_2_O_2_. Following three PBST washes, the samples were incubated in TSA-Plus tetramethylrhodamine (TMR) for 1 h. Double color fluorescence in situ hybridization was performed using TSA Plus fluorescein & TMR according to the manufacturer’s instruction (NEL756, PerkinElmer). The nuclei were stained using 4’-6-diamidino-2-phenylindole (DAPI) and embedded in ProLong Gold Antifade reagent (Invitrogen, Carlsbad, CA, USA). The slides were then mounted and photographed by Nikon Eclipse 50i fluorescence microscope (Tokyo, Japan). In this procedure, two antisense RNA probes were co-incubated in a single sample during the hybridization step, to develop red and green fluorescence (p-4) (Chi et al. 2017).

### Histology

The Atlantic salmon brain were fixed in Bouin’s fixative for 24 h and preserved in 70% ethanol. The samples were stained using hematoxylin and eosin (H&E) and sections were observed by a light microscope (NikonYS-100, Japan). Photographs were taken with a digital camera (Nikon coolpix-4500, Japan).

**Table 2.** Primers use for *in situ* hybridization.

Genes	Sequences
*AsMR*	F: 5’ CATCAACCGCTACTGCTACATCTGTCA 3’
* *	R: 5’ CACCTCTGTCTTCACCTTCCTCCTCA3’
*AsLR*	F:5’ GGGCACTGTTACTGAGGAGCGAATA 3’
* *	R:5’ CGACCACTCTACAACCAGGGACCAC 3

Note: F: forward primer; R: reverse primer.

### Statistical analysis

All statistical analyses were performed using SPSS version 20.0. The results were presented as means ± standard deviation (SD) and compared using a one-way analysis of variance (ANOVA) followed by Tukey’s test. All assays were performed independently in triplicate.

## Results

### Location of *AsMR* and *AsLRa1a1* in the brain of Atlantic salmon

**Figure 1.**
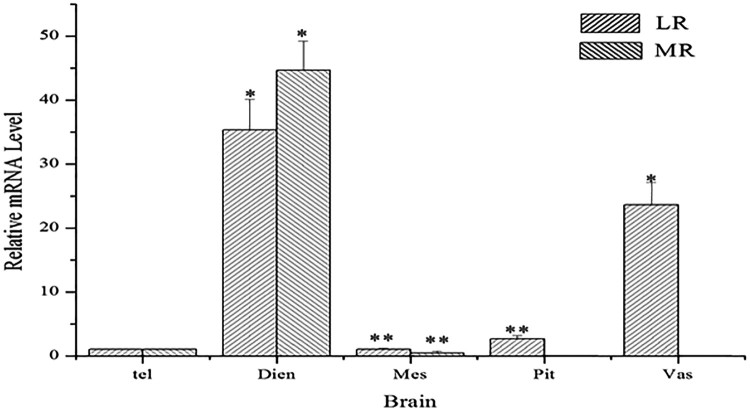
Distribution of *AsLRa1* and *AsMR* in the Atlantic salmon brain. Tel: telencephalon; Dien: diencephalon; Mes: mesencephalon; Pit: pituitary gland; Vas: saccus vasculosus.

**Figure 2.**
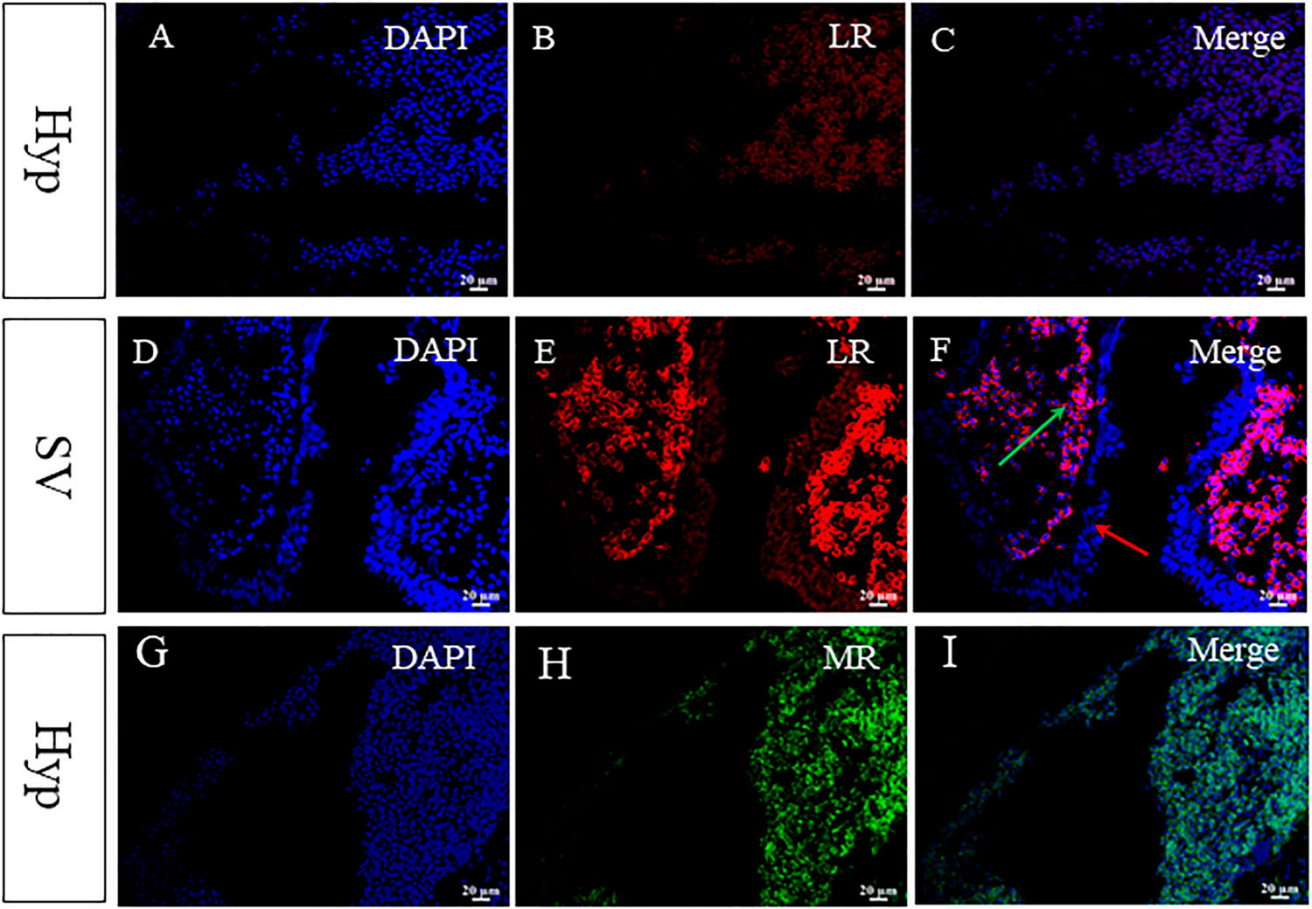
Expression of *AsLRa1* and *AsMR* in the hypothalamus and saccus vasculosus. (A–C) Expression pattern of *AsLRa1* in the hypothalamus of Atlantic salmon brain; (D, E) Expression pattern of *AsLRa1* in th saccus vasculosus of Atlantic salmon brain; (G–I) Location of *AsMR* mRNA in the hypothalamus using *in situ hybridization.* The green arrow indicates the cerebrospinal fluid-contacting cells; and the red arrow indicates the coronet cells. SV: saccus vasculosus; Hyp: hypothalamus.

The location of *AsMR* and *AsLRa1* were examined by quantitative real-time PCR with *β-actin* mRNA as a loading control. The results showed that *AsLRa1* was primarily expressed in the diencephalon, pituitary gland and SV, and *AsMR* were mainly expressed in the diencephalon in the Atlantic salmon brain (Figure 1). To confirm the precise location of *AsLRa1*, the diencephalon and SV were isolated to perform in situ hybridization. The results showed that both *AsLRa1* and *AsMR* transcripts were mainly expressed in the hypothalamus of the diencephalon (Figure 2(G–I). In the SV, the *AsLRa1* transcripts mainly appeared in the cerebrospinal fluid-contacting (CSF-c) cells (Figure 2(D–F). The histology of SV were shown in supply materials.)

### Expression pattern of *AsMR* in the different photoperiod in the hypothalamus of Atlantic salmon

**Figure 3.**
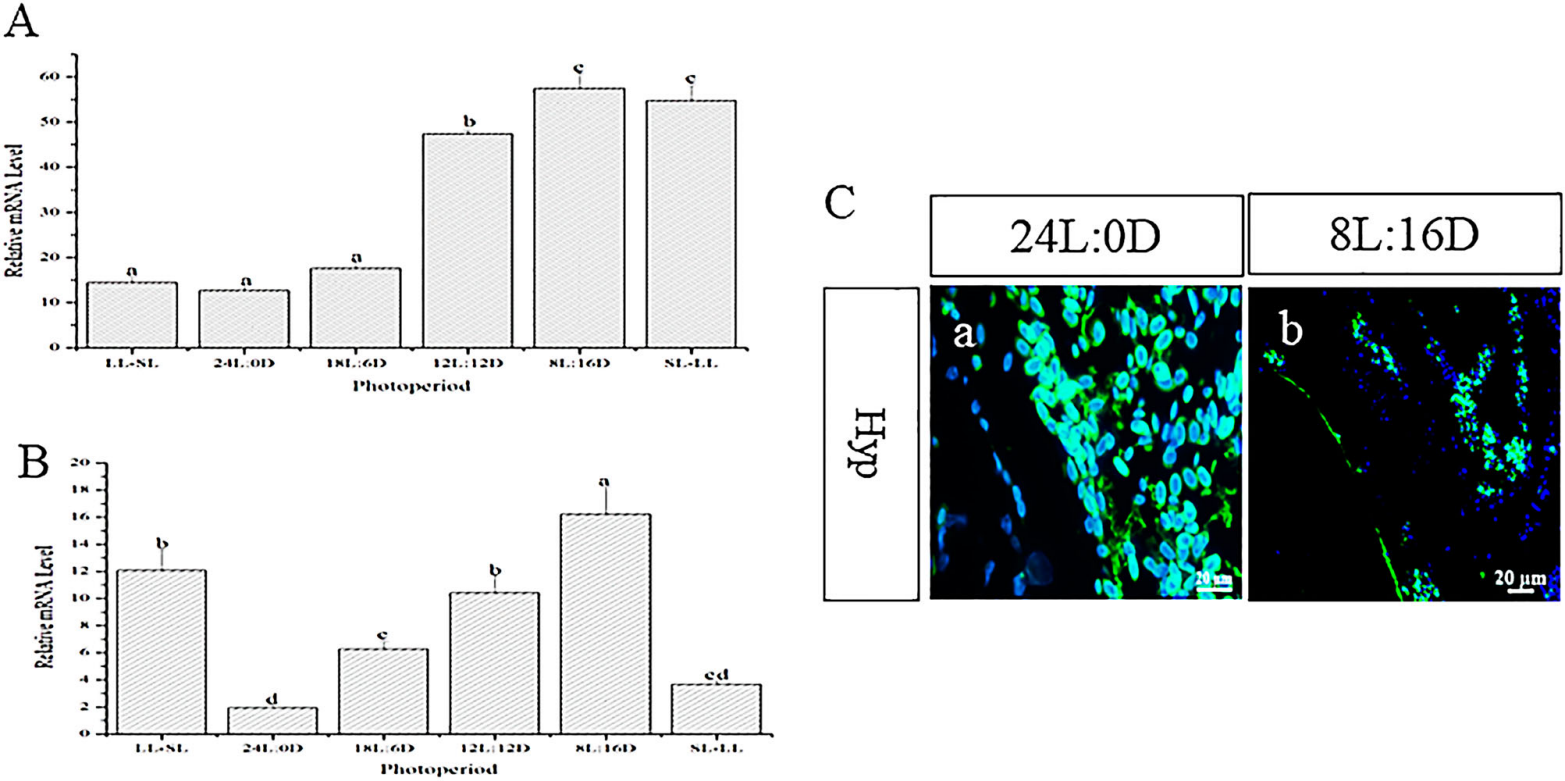
Expression pattern of *AsMR* transcripts in different photoperiods. (A): Expression pattern of *AsMR* transcripts in different photoperiod at the early stage of the experiment; (B): Expression pattern of *AsMR* transcripts in different photoperiod at the end of the experiment. Different letters indicate statistical significance at *p* < 0.05; (C): The expression of *AsMR* in the Atlantic salmon hypothalamus in long photoperiod (a) and short photoperiod (b) are assayed using *in situ* hybridization

Expression of *AsMR* in the hypothalamus could be influenced by photoperiod. At the beginning of the experiment, the *AsMR* transcripts levels were lowest in the 24L:0D group, followed by the LL-SL group. The expression level of *AsMR* transcripts was highest in the 8L:16D group followed by the SL-LL group (Figure 3(A)) At the end of the experiment, the lowest level of *AsMR* transcripts appeared in the 24L:0D and SL-LL groups and the highest level in the 8L:16D and LL-SL groups (Figure 3(B)). Furthermore, the expression of *AsMR* in the hypothalamus were detected using *in situ* hybridization, the results show that the expression levels of *AsMR* under long photoperiod (24L:0D)transcripts were significantly higher than short photoperiod (8L:16D) (Figure 3(C)).

### Expression pattern of *AsLRa1* in different photoperiods in the hypothalamus and SV of Atlantic salmon

**Figure 4.**
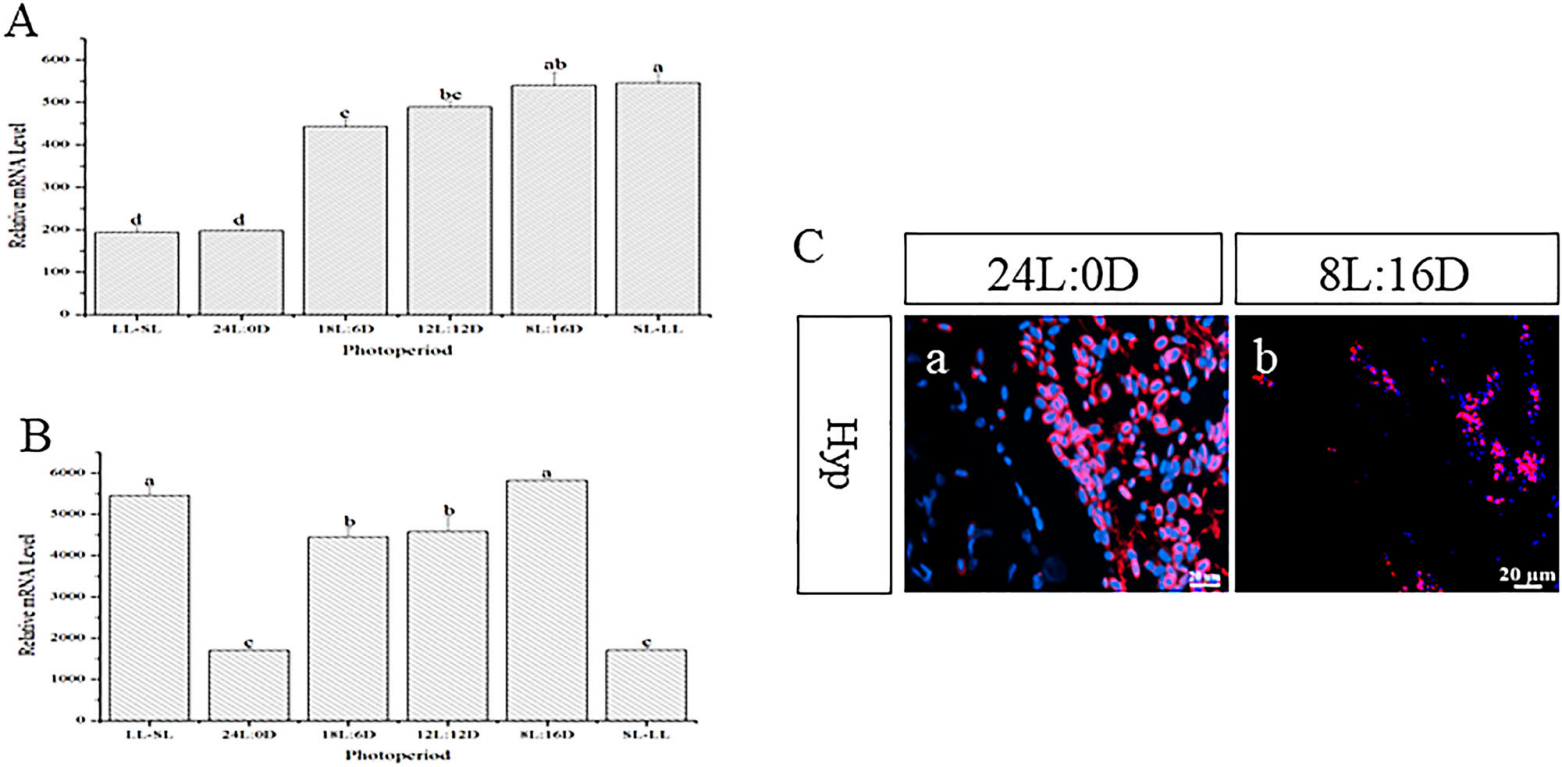
Expression pattern of *AsLRa1* transcripts in the Atlantic salmon hypothalamus in different photoperiods. (A): Expression pattern of *AsLRa1* transcripts in different photoperiods at the early stage of the experiment; (B): Expression pattern of *AsLRa1* transcripts in different photoperiods at the end of the experiment. Different letters indicate statistical significance at *p* < 0.05. (C): The expression of *AsLRa1* in the Atlantic salmon hypothalamus in long photoperiod (a) and short photoperiod (b) are assayed using *in situ* hybridization

**Figure 5.**
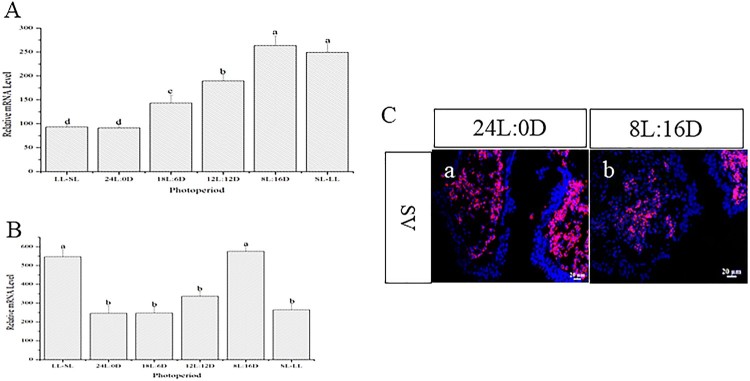
Expression pattern of *AsLRa1* transcripts in the Atlantic salmon SV in different photoperiod. (A): Expression pattern of *AsLRa1* transcripts in different photoperiods at the early stage of the experiment; (B): Expression pattern of *AsLRa1* transcripts in different photoperiods at the end of the experiment. Different letters indicate statistical significance at *p* < 0.05. (C): The expression of *AsLRa1* in the Atlantic salmon SV in long photoperiod (a) and short photoperiod (b) are assayed using *in situ* hybridization

Since *AsLRa1* was mainly expressed in the hypothalamus and SV, we examined the expression pattern of *AsLRa1* in the hypothalamus and SV under different photoperiod. The results showed that photoperiod affected the expression of *AsLRa1* in the hypothalamus and SV. In the hypothalamus, *AsLRa1* transcripts were lowest in the 24L:0D and the LL-SL photoperiod groups (Figure 4(A)) at the early stage of the experiment. At this time, these two treatments had the longest photoperiod. At the end time of the experiment, the lowest *AsLRa1* transcripts levels were in the 24L:0D group followed by the SL-LL group (Figure 4(B)). The expression of *AsLRa1* in the hypothalamus were also detected using *in situ* hybridization, the results show that the expression levels of *AsLRa1* under long photoperiod (24L:0D)transcripts were significantly higher than short photoperiod (8L:16D) (Figure 4(C)). The expression pattern of *AsLRa1* in the SV is similar to the hypothalamus. However, the expression level of *AsLRa1* in the SV is lower than that in the hypothalamus (Figure 5(A,B)). And the expression levels of *AsLRa1* in SV under long photoperiod (24L:0D) transcripts were higher than short photoperiod (8L:16D) (Figure 5(C)).

### Food intake of Atlantic salmon in different photoperiods

**Figure 6.**
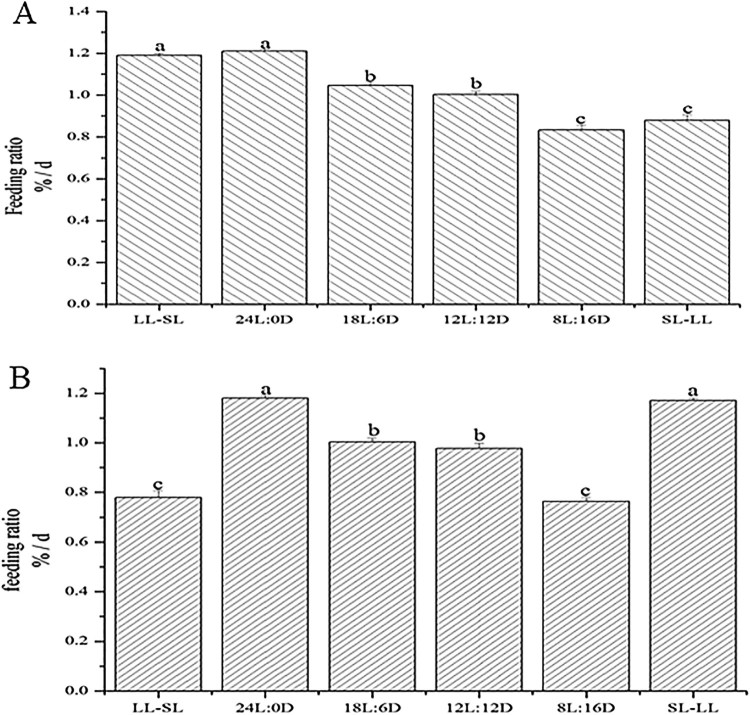
Feeding ratio (FR) of Atlantic salmon in different photoperiods. (A): FR of Atlantic salmon under different photoperiods at the early stage of the experiment; (B): The FR of Atlantic salmon under different photoperiods at the end of the experiment. Different letters indicate statistical significance at *p* < 0.05.

The daily FR could be affected by photoperiod. At the early stage of the experiment, the higher FR appeared in the 24L:0D photoperiod (1.21%/day), followed by the LL-SL photoperiod group(1.19%/day) (Figure 6(A)). At the end of the experiment, the highest FR were found in the 24L:0D photoperiod group (1.18%/day) and SL-LL photoperiod group (1.17%/day), (the photoperiod was longest at the end of experiment) (Figure 6(B)).

## Discussion

Leptin exerts its appetite-inhibiting effects by acting on the appetite control within the hypothalamus, and its can regulates a crowd of neuropeptides which located in hypothalamic (Crown et al. 2007). Furthermore, some researchers have found that leptin may regulate the expression and secretion of pituitary hormones in pituitary directly, by activating leptin receptor (Lloyd et al. 2001; Sone et al. 2001). However, whether leptin/leptin receptor were controlled by photoperiod is still unclear.

In recent years, Angotzi et al found a novel leptin receptor duplicate in Atlantic salmon, named LepRA1and LepRA2, and they suggested that leptin’s roles as modulator of nutritional status in Atlantic salmon might be governed by distinct genetic evolutionary processes and distinct functions between the paralogs, however, what’s the role of two paralogs are still not clear (Angotzi et al. 2016; Yan et al. 2017).

In this experiment, the fish were reared in a RAS, which provide a nearly consistent environment for Atlantic salmon. Furthermore, RAS enables the study of the effects of the photoperiod on growth, independent of other environmental factors. In our previous study, we found that photoperiod also promoted somatic growth of Atlantic salmon reared in RAS (in press). In order to investigate the mechanism behind the effect of photoperiod on the growth of Atlantic salmon, we examined the relationship between photoperiod and *AsLRa1*.

Firstly, we determined the location of *AsLRa1* in the Atlantic salmon brain. The results showed that *AsLRa1* transcripts were mainly expressed in the hypothalamus and SV. This indicated that leptin may play a role in both the hypothalamus and SV of Atlantic salmon. The SV is a circumventricular organ of the hypothalamus and unique to fish, the function of SV is still unclear. A recent study found that the SV of masu salmon is a sensor of seasonal changes in day length (Nakane et al. 2013). In order to investigate whether the SV is an organ that can regulate seasonal growth via the LR in Atlantic salmon, the changes in expression of *AsLRa1* under different photoperiod treatments were examined. The changes in *AsLRa1* transcript levels in the SV under the different photoperiod treatments are similar to the changes in the hypothalamus. However, the expression levels of *AsLRa1* are lower than those in the hypothalamus. In our previous paper, we found that SV is an organ which can regulate reproduction via photoperiodic signals (Chi et al. 2017). Here, we found that the LR in Atlantic salmon SV share the same pattern with kisspeptin receptor. So we suggest that in the Atlantic salmon, the SV also assists in regulating growth via photoperiodic signals besides the hypothalamus.

Secondly, the expression pattern of *AsLRa1* in the hypothalamus and SV of Atlantic salmon in different photoperiods was examined by qPCR. Expression of *AsLRa1* was affected by photoperiod, and long photoperiod suppressed the expression of *AsLRa1* both in the hypothalamus and SV. Melatonin is the most important internal timekeeping molecule that is involved in the control of daily variations of locomotor activity, such as growth and reproduction in fish (Boeuf and Falcon 2001; Falcon et al. 2003; Zachmann et al. 1992). In order to confirm whether *AsLRa1* could be affected by photoperiod, we also examined the expression of *MR* of Atlantic salmon in the hypothalamus under different photoperiods. The results showed that the expression of *AsMR* in the hypothalamus had a similar expression pattern in the different photoperiods as *AsLRa1.* Furthermore, we found that both *AsMR* and *AsLRa1* were expressed in the same cells in hypothalamus. So we speculated that the rhythm of AsLRa1 might be regulated by photoperiod via melatonin.

After confirming the relationship between photoperiod and *AsLRa1*, we investigated whether food intake was affected by photoperiod. The results showed that the daily FR was affected by photoperiod. FR of Atlantic salmon was higher during a long photoperiod compared to short photoperiod. Meanwhile, the expression pattern of *AsLRa1* is contrasted with the pattern of food intake. So we speculated that in the long photoperiod, the expression *AsLRa1* was inhibited, which increased the appetite of Atlantic salmon, and led to higher growth rate. In the short photoperiod, the *AsLRa1* transcript levels were higher, and the *AsLRa1* would induce higher expression of leptin to suppress appetite (de Git and Adan 2015). Then Atlantic salmon would have higher growth rate under the long photoperiod and show lower growth rates under the short photoperiod. In conclusion, we found that photoperiod regulated growth of Atlantic salmon may occur via the LRs receptor both in the hypothalamus and saccus vasculosus.
